# Dr. Richard J. Dunglison

**Published:** 1901-04

**Authors:** 


### OBITUARY NOTES.

Dr. Richard J. Dunglison, of Philadelphia, died at his home March 4, 1901, aged 66 years. Dr. Dunglison was the son of Dr. Robley Dunglison, the editor of Dunglison's Dictionary, and of which he, himself, was editor during many editions. He was also editor of Dunglison's College and Clinical Record, and served as treasurer for the American Medical Association for several years.

